# Transforming Marble Waste into High-Performance, Water-Resistant, and Thermally Insulative Hybrid Polymer Composites for Environmental Sustainability

**DOI:** 10.3390/polym12081781

**Published:** 2020-08-09

**Authors:** Payal Bakshi, Asokan Pappu, Ravi Patidar, Manoj Kumar Gupta, Vijay Kumar Thakur

**Affiliations:** 1 Academy of Scientific and Innovative Research (AcSIR), CSIR—Advanced Materials and Processes Research Institute, Bhopal, Madhya Pradesh 462026, India; bakshi.payal@gmail.com (P.B.); manojampri@gmail.com (M.K.G.); 2 CSIR—Advanced Materials and Processes Research Institute, Bhopal, Madhya Pradesh 462026, India; ravipatidar213@gmail.com; 3 Biorefining and Advanced Materials Research Center, Scotland’s Rural College (SRUC), Kings Buildings, Edinburgh EH9 3JG, UK

**Keywords:** high-strength composite, thermal conductivity, recycling, marble waste particulates, low-density materials, injection-molded specimen

## Abstract

Marble waste is generated by marble processing units in large quantities and dumped onto open land areas. This creates environmental problems by contaminating soil, water, and air with adverse health effects on all the living organisms. In this work, we report on understanding the use of calcium-rich marble waste particulates (MPs) as economic reinforcement in recyclable polypropylene (PP) to prepare sustainable composites via the injection molding method. The process was optimized to make lightweight and high-strength thermally insulated sustainable composites. Physicochemical, mineralogical, and microscopic characterization of the processed marble waste particulates were carried out in detail. Composite samples were subsequently prepared via the injection molding technique with different filler concentrations (0%, 20%, 40%, 60%, and 80%) on weight fraction at temperatures of 160, 180, and 200 °C. Detailed analysis of the mechanical and thermal properties of the fabricated composites was carried out. The composites showed a density varying from 0.96 to 1.27 g/cm^3^, while the water absorption capacity was very low at 0.006–0.034%. Marble waste particulates were found to considerably increase the tensile, as well as flexural, strength of the sustainable composites, which varied from 22.06 to 30.65 MPa and 43.27 to 58.11 MPa, respectively, for the molding temperature of 160 °C. The impact strength of the sustainable composites was found to surge with the increment in filler concentration, and the maximum impact strength was recorded as 1.66 kJ/m^2^ with 20% particulates reinforcement at a molding temperature of 200 °C. The thermal conductivity of the particulates-reinforced sustainable composites was as low as 0.23 W m^−1^K^−1^ at a 200 °C molding temperature with 20% and 40% filler concentrations, and the maximum thermal conductivity was 0.48 W m^−1^K^−1^ at a 160 °C molding temperature with 80% filler concentration. Our findings have shown a technically feasible option for manufacturing a lightweight composite with better mechanical and thermal properties using marble waste particulates as a potential civil infrastructural material.

## 1. Introduction

Marble processing units generate huge quantities of waste during different maneuvers such as cutting and polishing processes. It is produced in a slurry form, carried by the drainage system, and is discarded in open land areas, which, on drying, creates severe environmental issues. This accumulated marble waste contaminates water and air, and has harmful effects on plants, animals, and human health [1,2,3,4]. About 200 million tons of marble waste by marble processing industries, as a waste powder or sludge, has been produced worldwide. About 16 million tons of this waste has been created in India alone annually [5,6,7,8]. These marble processing industries involve mines and processing units for manufacturing tiles, blocks, decorative articles, waste disposal areas, and other activities [9,10]. Proper handling and management of marble wastes are necessary to protect the environment from the soil, water, and air contamination [6,11].

The effective use of marble waste particulates in the development of innovative advanced materials will serve the purpose of protecting the earth and becoming clean and green. Sustainable polymer composites may be one of the finest solicitations to use waste marble for replacing the traditional fillers, clay, and other conventional materials [5,12,13]. Use of mineral fillers like marble waste particulates in the manufacturing of polymer composites is generally cost-effective. The other properties of such fillers are usually particle shape, size, and particle size distribution [14]. Moreover, polypropylene (PP), used as the matrix in this study, is a popular polymer with good processability, dimensional stability, low price, chemical resistance, stabilized properties, transparency, and higher impact strength. PP has been reported to be an appropriate polymer material for reinforcing blending and filling [15,16,17,18].

Researchers have attempted to use marble waste particulates in paver blocks [19], soil stabilization [11,20], cement-based adhesive mortar [21], and as a replacement of conventional natural aggregates in concrete [4,8,22,23,24,25]. Attempts have also been made to use marble waste for manufacturing cementitious roofing tiles [26], preparing polymer concrete with unsaturated pre-accelerated polyester resin [27], a cement-based composite material with expanded perlite and tragacanth (plant resin) [28], composite bricks [2,6,29], artificial stones, unsaturated polyester resin, and epoxy resin as binders [30,31].

The appropriate combination of marble waste particulates with silica and carbon black was used as a potential reinforcing material in the manufacturing of hybrid composites using natural rubber as the matrix material [12]. Marble waste was used as filler with an ethylene-propylene-diene monomer (EPDM) with chloroprene rubber [32]. The addition of marble and granite waste powder was done with high-density polyethylene matrix to analyze the influence of filler particle size and its content on the thermal and mechanical properties [33]. Composite materials have been produced using PET (polyethylene terephthalate) bottles and marble dust [34]. Different ratios of fly ash, marble waste particulates, and polyester resin [35] were studied. Marble waste particulates in a polypropylene matrix with wood particulates [1] and magnesium hydroxide for making flame retardant composite material [36] were also analyzed. Polymer composites were fabricated using waste marble particulates with unsaturated polyester resin [37], epoxy resin [7,38], and polypropylene matrix [9]. However, as per the authors’ best knowledge, very few works have been reported on the use of marble waste particulates (MPs) as filler in polypropylene (PP) composites by the injection molding (at different temperatures) technique and their physical and mechanical characterization.

As the generation of industrial wastes such as marble waste causes economic and environmental problems, the potential use of marble waste particulates in developing hybrid polymer composites can be realized. In this work, we have made efforts to effectively reuse marble waste particulates (MPs) to develop a lightweight and high-strength sustainable hybrid polymer composite in a polypropylene (PP) system for different construction applications. To achieve this objective, physicochemical characterization of the received marble waste particulates was carried out by determining moisture content, particle size, density, energy-dispersive X-ray spectroscopy (EDS), specific gravity, pH, and electrical conductivity. Morphological and mineralogical analysis of marble waste particulates was carried out by state-of-the-art SEM and X-ray techniques. MP-PP composite samples were prepared by the injection molding technique with different filler concentrations of 0%, 20%, 40%, 60%, and 80% at injection molding temperatures of 160, 180, and 200 °C.

## 2. Materials and Methods

### 2.1. Materials

The marble waste particulates (MPs) used was collected from a marble processing unit in Udaipur, Rajasthan, India. Polypropylene (PP) purchased from Three Star Plastics, Grade No. AM 650 N was used as a binder in the injection molding machine. Marble waste particulates were heated at 110 °C for 24 h, cooled in desiccators, and then sieved through a BS 150 (105 µm) sieve. Granulated PP was used as a polymer matrix with sulfur vulcanization as the catalyst. Sulfur vulcanization resultedincross-linking thermoplastic, which is non-toxic, safe to handle, and rapidly curing at about 150 °C with no tendency to scorch. Moreover, it is non-volatile, prevents loss during mixing, and acts as effective reinforcing filler.

### 2.2. Fabrication of Composites

**Figure 1:**
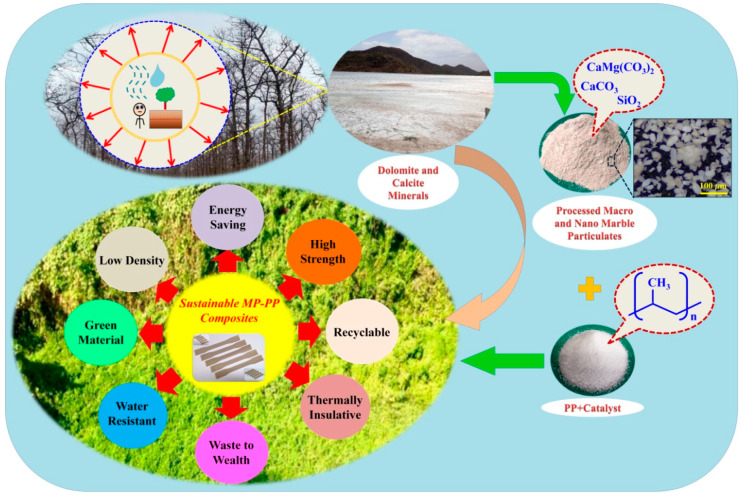
The schematic diagram for the fabrication of the composites.

For manufacturing hybrid polymer composites using marble waste particulates (MPs), these particulates were mixed with the polypropylene (PP), and composites were prepared using an injection molding machine (Milacron, Nova Servo 150, Batavia, OH, USA) at 160, 180, and 200 °C. Marble waste particulates as filler were used at concentrations of 0, 20, 40, 60, and 80 wt. % in the polypropylene (PP) system. A schematic diagram for the fabrication of the hybrid composite using marble waste particulates with polypropylene is shown in Figure 1. The dumping of marble waste (dolomite and calcite-rich) onto open land area has adverse effects on air, water, and all the living organisms including plants, animals, and human health. CaMg(CO_3_)_2_(dolomite), CaCO_3_(calcite) and SiO_2_(quartz) are the mineral phases confirmed by XRD analysis in marble waste used in this study. After processing the marble waste particulates (heating at 110 °C for 24 h and then cooled in desiccators and sieved through a BS 150/105 µm sieve), it was mixed with granular polypropylene (PP), and sustainable MP-PP composites were prepared using an injection molding machine at 160, 180, and 200 °C. After the experimental investigation carried out in the present study, it was found that the prepared sustainable composites were green materials manufactured by marble waste and were low density, energy-saving, high-strength, recyclable, thermally insulative, and water-resistant (Figure 1).

### 2.3. Measurement and Characterizations

The moisture content of the obtained marble waste particulate sample was determined as per IS: 2720 (Part II)—1973 (Reaffirmed 1997) by the oven drying method. At 110 ± 5 °C, the average water content of the received sample was determined [39]. The specific gravity of the obtained marble waste particulate sample was determined as per IS: 2720 (Part III/ Sec 1)—1980 (Reaffirmed 2002), and the bulk density was determined with 25 mL density bottles [40]. The particle size distribution of the waste marble particulate specimen was analyzed using a Laser Scattering Particle Size Distribution Analyzer Partica LA-950, HORIBA Scientific Northampton, UK). The pH of obtained marble waste particulates was determined by a LABMAN LMPH–12 pH meter (Chennai, India). The electrical conductivity of obtained marble waste particulates was determined by a LABMAN LMCM-20 conductivity meter (Chennai, India). Colored micrographs of marble waste particulates were obtained by an Olympus DSX 1000 Digital Microscope (Tokyo, Japan). Elemental and morphological analysis of marble waste particulates and fabricated composites were carried out by SEM-EDS on a JEOL/EO JCM-6000Plus Benchtop SED (Peabody, MA, USA). X-ray diffraction patterns of the received marble waste sample were obtained by Rigaku, MiniFlex II Desktop X-ray diffraction instrument (Tokyo, Japan).

ASTM D 792-13 was used to determine the density of MP-PP composites (Equation (1)). The water absorption of composites was analyzed using ASTM D 570-98 (Reapproved 2018). Distilled water was used to submerge each sample separately at 25 ± 2 °C for the specified time interval of 24 h to determine the water absorption of the composite as per the previous protocol (Equation (2)) [1,41,42,43].

(1) $$Density\;\left(g/cm^{3}\right)=\frac{Mass}{Volume}$$

(2) $$\;WaterAbsorption\;\left(\%\right)=\frac{W_{1}-W_{2}}{W_{2}}\;$$

where *W*_1_ and *W*_2_ are the wet weight and conditioned weight of the sample.

The ASTM D 638-14 method was used to determine the tensile strength of injection-molded sustainable composite/PP specimens (Equation (3)). Samples were analyzed using a WDW-50Universal testing machine (Jinan Testing Equipment IE Corporation, Jinan, China), using a load cell of 50 kN and a gauge length of 50 mm with a 5 mm/min cross-head speed [1,14,41,44]. Flexural strength of injection-molded specimens was analyzed using ASTM D 790-17, using an LRX plus Universal testing machine (Ametek, Lloyd Instruments, Bognor Regis, UK), using a load cell of 5 kN having a 53 mm span and a 5 mm/min cross-head speed (Equation (4)) [1,14,41,45]. For impact testing, the notch was cut in injection-molded rectangular specimens using a Model 899 Impact Specimen Notcher for Plastics (Tinius Olsen, Horsham, PA, USA). The Izod impact testing was done employing ASTM D 256-10 (Reapproved 2018), using an impact tester (Model Impact 104, Tinius Olsen, Horsham, PA, USA), with a pendulum weight of 0.459 kg, pendulum radius of 334.963 mm, height of pendulum of 612.226 mm, and potential energy of 2.76 J at room temperature (Equation (5)) [14,42].The tensile modulus (TM) and flexural modulus (FM) were determined as per Equations (6) and (7), respectively. Five observations were taken and their mean values with standard deviation (Equation (8)) are reported. The thermal conductivity of MP-PP composites/PP had been measured by a Box-type probe PD-11N analyzer (KEM, Quick Thermal Conductivity Meter, QTM 710, Kyoto, Japan) at room temperature (Equation (9)).

(3) $$TensileStrength\left(\sigma_{u}\right),\;MPa=W/A_{0}$$

where *W* is breaking load and *A*_0_ is the original cross-sectional area.

(4) $$FlexuralStrength\left(\sigma_{f}\right),MPa=3PL/2bd^{2}$$

where, at a given point, *P* represents the load, *L* is the support span, *b* is the sample width, and *d* is the sample depth.

(5) $$ImpactStrength\left(I_{s}\right),\;J/m^{2}=E_{s}/bd$$

where *E*_s_ is the breaking energy, *b* is the sample width, and *d* is the sample depth.

(6) TensileModulus(E), GPa=σ/ε

where *σ* is the tensile stress and *ɛ* is the tensile strain.

(7) $$FlexuralModulus\left(E_{B}\right),\;GPa=L^{3}m/4bd^{3}$$

where *L* represents span, *m* represents the slope of the load-deflection curve, and *b* and *d* are the width and depth of the sample, respectively.

(8) $$StandardDeviation\left(s\right)=\sqrt{\sum X^{2}-n{\bar{X}}^{2}}/\surd \left(n-1\right)$$

where *X* is the value of a single observation, *n* is the number of observations, and $\bar{X}$ is the arithmetic mean of the set of observations.

(9) $$\lambda =K.R.I^{2}\frac{\ln\left(t_{1}/t_{2}\right)}{\left(T_{1}-T_{2}\right)}-H$$

where λ is the thermal conductivity, K and *H* are probe constants, R is the electric resistance of the probe heater, I is the heater current, t_1_ and t_2_ are the times after heating started, and T_1_ and T_2_ are the temperatures at t_1_ and t_2_.

## 3. Results and Discussion

### 3.1. Physicochemical Properties of Marble Waste Particulates

**Figure 2:**
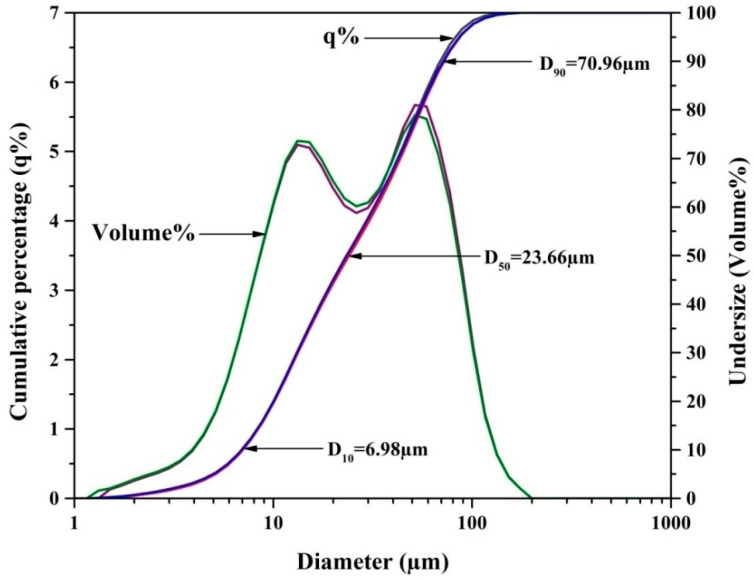
Particle size distribution curve of marble waste particulates.

The marble waste particulates presented were characterized for their physicochemical properties by standard methods. Table 1 depicts the characterization results. For all the studied properties, three observations were taken, and the mean values with standard deviation are reported. The collected marble waste particulate sample shows a silt texture with white color. The moisture content, bulk density, and specific gravity of the marble waste particulates were found to be 1.80 ± 0.09%, 1.39 ± 0.01 g/cm^3^, and 2.58 ± 0.03, respectively. The mean particle size of waste particulates was found to be 32.62 ± 0.61 µm. By the particle size distribution curve, it was observed that in the marble waste particulates used, 90% of the total volume had a diameter smaller than 70.96 µm (D_90_), 50% of the total volume had a diameter smaller than 23.66 µm (D_50_), and 10% of the total volume had a diameter smaller than 6.98 µm (D_10_). Particle size analysis depicts that the particle size was in the range of 6.98 to 70.96 µm (D_10_ = 6.98 µm, D_50_ = 23.66 µm, and D_90_ = 70.96 µm) (Figure 2). The pH of marble waste particulates was found to be 6.58 ± 0.26. An electrical conductivity of 0.065 ± 0.02 dS/m was observed at room temperature.

The chemical composition of marble waste particulates studied by EDS analysis is shown in Table 2. The mass% values are reported for each element. The results of elemental analysis by EDS shows the existence of oxygen, carbon, calcium, magnesium, aluminum, silicon, manganese, sodium, iron, potassium, and titanium.

### 3.2. Particle Size Analysis

The obtained marble waste particulate sample’s particle size distribution was determined by a dynamic laser scattering analyzer, and Figure 2 represents the curve for particle size distribution. The curve shows the cumulative (q %) and undersize concerning the particle size of waste marble particulates. The outcomes of the study indicated that the marble waste particulate sample had fine particle sizes. The results indicate that 90% of the total volume had a smaller diameter than 70.96 µm (D_90_), 50% of the total volume had a smaller diameter than 23.66 µm (D_50_), and 10% of the total volume had a smaller diameter than 6.98 µm (D_10_). The mean particle size of the marble waste particulate sample was 32.62 ± 0.61 µm. Undersize (volume %) shows a broad distribution extending from a minimum of 1.318 to a maximum of 174.616 µm.

### 3.3. Mineralogical Analysis

**Figure 3:**
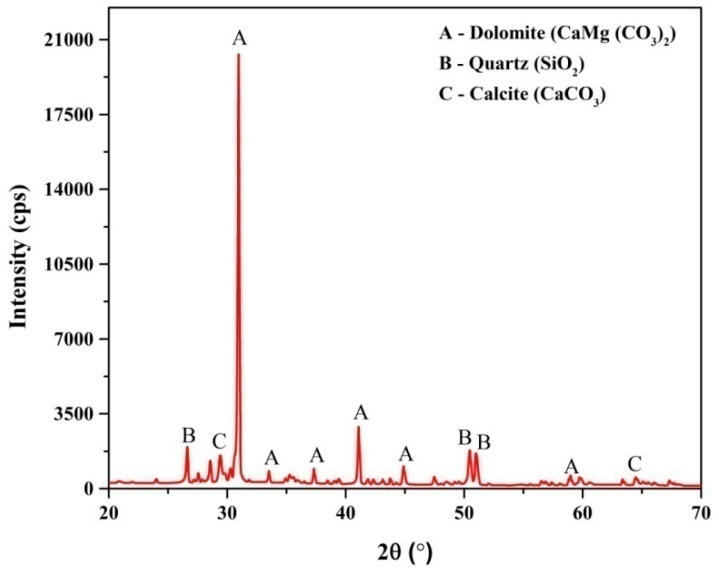
XRD pattern of marble waste particulates.

The mineralogical study of marble waste particulates was carried out using X-ray diffraction analysis in the 2θ range of 20°–70°. Figure 3 shows the XRD pattern of marble waste particulates. To identify the mineral phases, all diffraction peaks were matched with the JCPDS card numbers. The XRD study mainly confirmed the presence of the dolomite (CaMg(CO_3_)_2_) phase (JCPDS card no. 84-1208), quartz (SiO_2_) phase (JCPDS card no. 86-1629), and calcite (CaCO_3_) mineral phases (JCPDS card no. 05-0586). It is also clear that the marble waste particulate sample has a crystal structure and the concentration of the dolomite phase is higher than other minerals.

### 3.4. Morphological Analysis of Marble Waste Particulates

**Figure 4:**
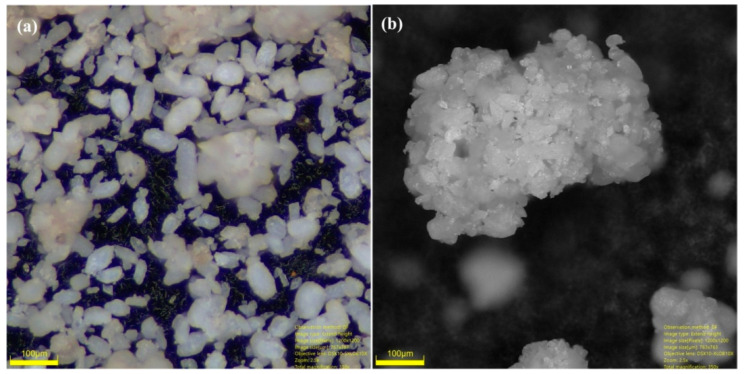
Digital micrographs of (**a**) irregular morphology and (**b**) cluster of marble waste particulates.

The morphology and size of marble waste particulates were investigated by the digital microscopy technique. Digital micrographs of the marble waste particulate sample are shown in Figure 4a,b. The micrographs indicate a cluster of micro-sized particles with irregular morphology including rhombic structures and cubic shape. The size of particles is in the range of 7 to 80 µm. To remove the moisture content in marble waste, the samples were thermally annealed at 110 °C and sieved through a BS 150 (105 µm) sieve.

### 3.5. Density and Water Absorption Capacity of the Composites

**Figure 5:**
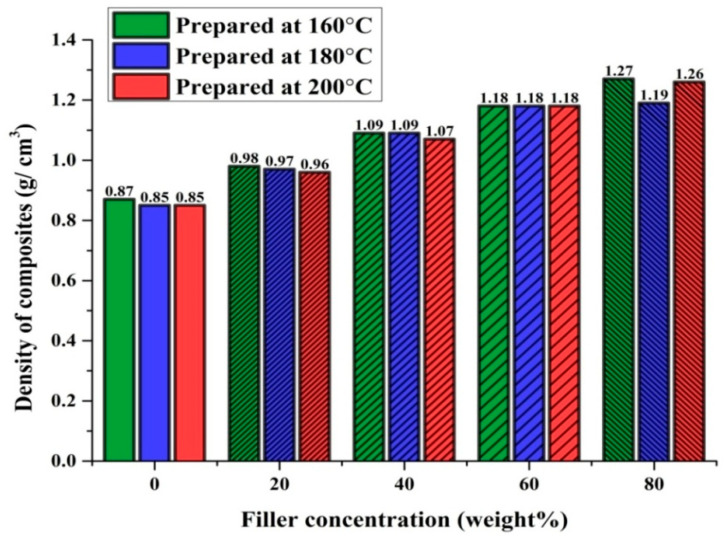
The density of MP-polypropylene (PP) composites/PP.

Density, as well as water absorption, studies were carried out for pure PP and marble waste particulates-based polypropylene composites. Table 3 encapsulates the measurements results. To determine the density of the fabricated composite samples as per Equation (1), three observations were taken, and their mean value is presented in Figure 5.

The density of pure polypropylene (PP) was found to be 0.87 g/cm^3^ at 160 °C and 0.85 g/cm^3^ at 180 and 200 °C. MP-PP composites prepared at 160 °C with 20, 40, 60, and 80 wt. % filler concentrations showed densities of 0.98, 1.09, 1.18, and 1.27 g/cm^3^, respectively. MP-PP composites prepared at 180 °C with 20, 40, 60 and 80 wt. % filler concentrations showed densities of 0.97, 1.09, 1.18, and 1.19 g/cm^3^, respectively. MP-PP composites prepared at 200 °C with 20, 40, 60, and 80 wt. % filler concentrations showed densities of 0.96, 1.07, 1.18, and 1.26 g/cm^3^, respectively.

It was observed that the density of pure PP slightly decreased with an increase in molding temperature and the density of MP-PP composites increased with the increment in the concentration of the filler [16,46]. For the composites fabricated with 60 wt. % marble waste particulates in the PP system, the density was 1.18 g/cm^3^ for all the injection molding temperatures of 160, 180, and 200 °C. The density of the MP-PP composite was a minimum of 0.96 g/cm^3^ at a 200 °C molding temperature with a 20 wt. % filler concentration and a maximum of 1.27 g/cm^3^ at a 160 °C molding temperature with a 80 wt. % filler concentration.

**Figure 6:**
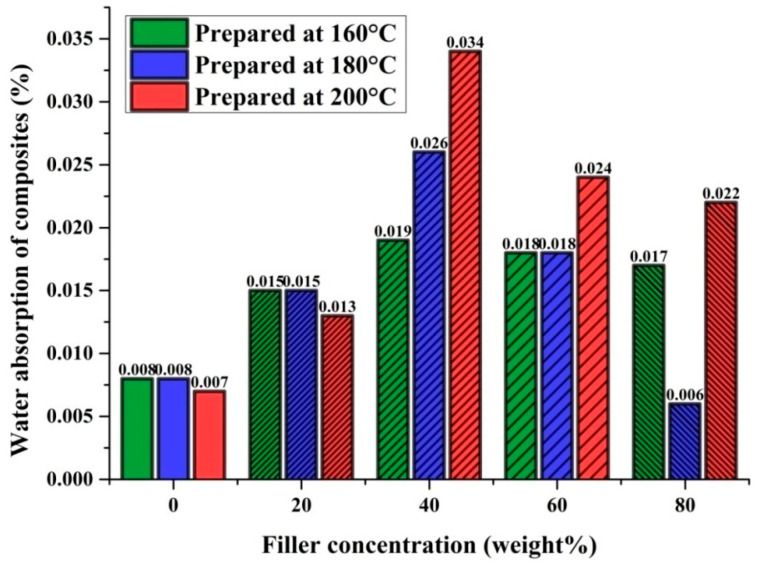
Water absorption of MP-PP composites/PP.

To determine the water absorption of the fabricated composite samples as per Equation (2), three observations were taken, and their mean value is presented in Figure 6.

Water absorption of 0.008% was observed for pure PP samples fabricated at 160 and 180 °C injection molding temperatures. At a 200 °C molding temperature, pure PP samples showed 0.007% water absorption. MP-PP composites prepared at 160 °C with 20, 40, 60, and 80 wt. % filler concentrations showed water absorptions of 0.015, 0.019, 0.018, and 0.017%, respectively. MP-PP composites prepared at 180 °C with 20, 40, 60, and 80 wt. % filler concentrations showed water absorptions of 0.015, 0.026, 0.018, and 0.006%, respectively. MP-PP composites prepared at 200 °C injection molding temperature with 20, 40, 60, and 80 wt. % filler concentrations showed water absorptions of 0.013, 0.034, 0.024, and 0.022%, respectively. The results indicated that with an increase in filler concentration up to 40%, the water absorption increases, and beyond 40% filler concentration, this decreases in the case of all the injection molding temperatures of 160, 180, and 200 °C [33,46]. The water absorption of the MP-PP composite was a minimum of 0.006% at a 180 °C molding temperature with 80 wt. % filler concentration and a maximum of 0.034% at a 200 °C molding temperature with 40 wt. % filler concentration. It was observed that the developed MP-PP composites showed very low water absorption of 0.006–0.034%. One of the focuses in the present study is to develop and understand the performance of the marble waste particulates-fortified polypropylene composite as a better alternative to wood–plastic composites (WPC). Findings of this study revealed that, as compared to wood–plastic composites (WPC), the water absorption is lower in the case of this MP-PP composite. Moreover, traditional materials such as wood, particleboard, or plywood exhibit higher water absorptions than the results achieved in the present study, which shows that the durability of MP-PP composites under different weathering conditions would not affect the composites’ performance and their service life. The results of water absorption studies showed that marble waste particulates can be potential filler with the polypropylene system for the development of waterproof hybrid polymer composites for multiple applications.

### 3.6. Mechanical Properties of Composites

**Figure 7:**
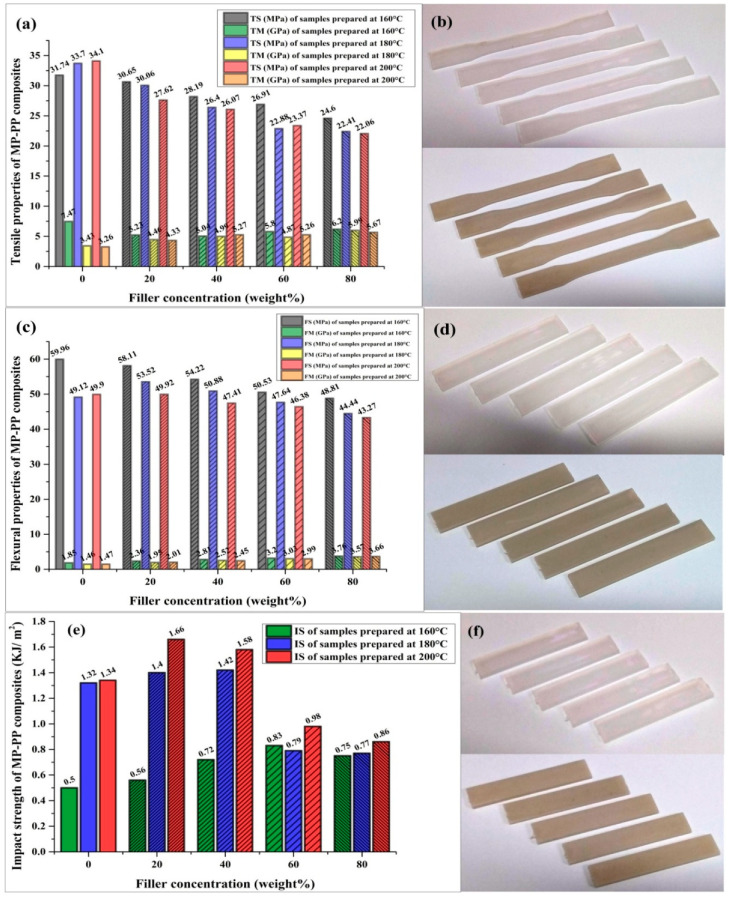
Mechanical properties of MP-PP composites: (**a**) Tensile strength and modulus, (**b**) tensile test specimens, (**c**) flexural strength and /modulus, (**d**) flexural test specimens, (**e**) impact strength, and (**f**) impact test specimens.

The mechanical properties of injection-molded pristine PP and marble waste particulate polymer composites with filler concentrations of 20, 40, 60, and 80 wt. % in a PP system at injection molding temperatures of 160, 180, and 200 °C were determined (Figure 7). The tensile/flexural/impact strength of fabricated composites (Figure 7a–f, respectively) was determined as per Equations (3)–(5), respectively, with the tensile modulus (TM) and flexural modulus (FM) as per Equations (6) and (7), respectively. The tensile, flexural, and impact test specimens of pure PP, PP with different concentration of marble waste particulates with their properties are shown in Figure 7. Five observations were taken for each studies and their mean values with standard deviation are reported the results are summarized in Table 3.

**Figure 8:**
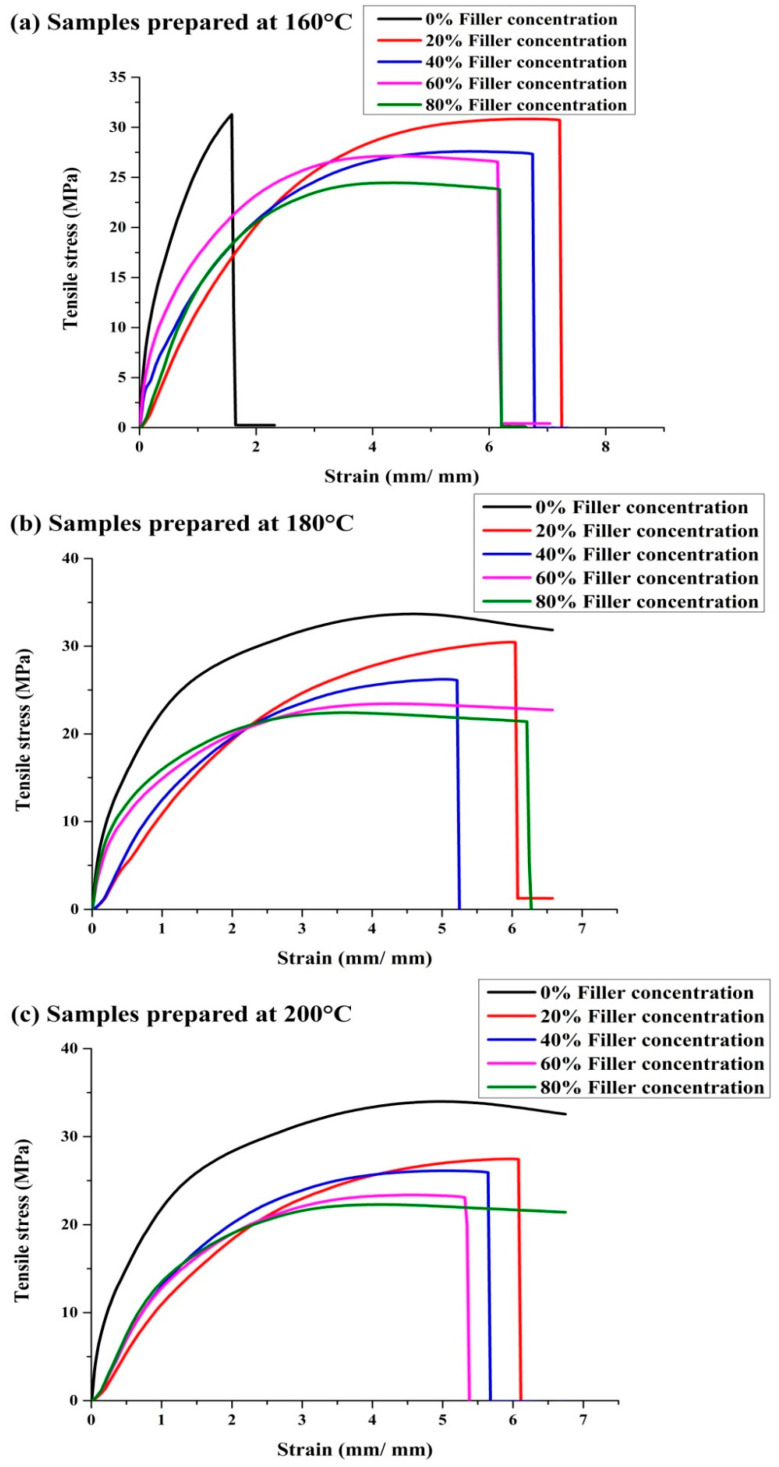
Stress–strain behavior of MP-PP composites/PP prepared at (**a**) 160, (**b**) 180, and (**c**) 200 °C.

The tensile strength of pristine PP and marble waste particulate (MP)-filled polymer composite samples (Figure 7b) fabricated by the injection molding process in the polypropylene (PP) system was determined as per the standard method. The tensile strengths of 0, 20, 40, 60, and 80 wt. % filler-content polymer composite samples fabricated at an injection molding temperature of 160 °C were found to be 31.74, 30.65, 28.19, 26.91, and 24.6 MPa, respectively. The tensile strengths of 0, 20, 40, 60, and 80 wt. % filler-content polymer composite samples fabricated at an injection molding temperature of 180 °C were found to be 33.7, 30.06, 26.4, 22.88, and 22.41 MPa, respectively. The tensile strengths of 0, 20, 40, 60 and 80 wt. % filler-content polymer composite samples fabricated at an injection molding temperature of 200 °C were found to be 34.1, 27.62, 26.07, 23.37, and 22.06 MPa, respectively. It is observed that the tensile strength of the fabricated sustainable polymer composite samples decreases with the increment in filler concentration and injection molding temperature [1], whereas the tensile strength of pure PP samples increases with molding temperature (Figure 7a). The tensile strength of the MP-PP composite is a maximum of 30.65 ± 0.99 MPa at a 160 °C molding temperature with 20 wt. % filler concentration (Table 3). The stress–strain behavior of fabricated composite samples prepared at injection molding temperatures of 160, 180, and 200 °C is displayed in Figure 8a–c, respectively. The stress-strain curves of MP-PP composites of different filler concentrations have been found to show the ductile failure of tested samples (Figure 8).

The flexural strength of pristine PP and marble waste particulate (MP)-filled polymer composites (Figure 7d) fabricated by the injection molding technique in the polypropylene (PP) system was also determined. The flexural strengths of 0, 20, 40, 60, and 80 wt. % filler-content polymer composites fabricated at an injection molding temperature of 160 °C were found to be 59.96, 58.11, 54.22, 50.53, and 48.81 MPa, respectively. The flexural strengths of 0, 20, 40, 60, and 80wt. % filler-content polymer composites fabricated at an injection molding temperature of 180 °C were found to be 49.12, 53.52, 50.88, 47.64, and 44.44 MPa, respectively. The flexural strengths of 0, 20, 40, 60, and 80 wt. % filler-content polymer composites fabricated at an injection molding temperature of 200 °C were found to be 49.90, 49.92, 47.41, 46.38, and 43.27 MPa, respectively. It has been observed from the study that the flexural properties of fabricated composite samples decrease with the increment in the filler concentration for samples prepared at a 160 °C injection molding temperature, whereas the flexural strength of the MP-PP composite samples increases for a 20 wt. % filler concentration at 180 and 200 °C molding temperatures [1,34,35],and beyond 20%, it again decreases but is higher than those of pure PP samples up to a 40 wt. % filler concentration for a 180 °C molding temperature (Figure 7c). This reduction in tensile and flexural strength with filler content may be due to the agglomeration phenomenon of filler particles in the PP matrix because of finer particle sizes [9,14,33]. The flexural strength of the MP-PP composite is a maximum of 58.11 ± 0.68 MPa at a 160 °C molding temperature with 20 wt. % filler concentration. The tensile and flexural modulus of the fabricated polymer composite samples is reported with the variation in filler concentration and fabrication temperature in Table 3.

The impact strength of pristine PP and marble waste particulate (MP)-filled polymer composite samples (Figure 7f) fabricated by the injection molding process in the polypropylene (PP) system was determined. The impact strengths of 0, 20, 40, 60, and 80 wt. % filler-concentration polymer composite samples fabricated at an injection molding temperature of 160 °C were found to be 0.50, 0.56, 0.72, 0.83, and 0.75 kJ/m^2^, respectively. The impact strengths of 0, 20, 40, 60, and 80 wt. % filler-concentration polymer composite samples fabricated at an injection molding temperature of 180 °C were found to be 1.32, 1.40, 1.42, 0.79 and 0.77 kJ/m^2^, respectively. The impact strengths of 0, 20, 40, 60, and 80 wt. % filler-concentration polymer composite samples fabricated at an injection molding temperature of 200 °C were found to be 1.34, 1.66, 1.58, 0.98, and 0.86 kJ/m^2^, respectively. It is worth noting that the impact properties of the fabricated sustainable composites rise with the increment in filler concentration up to 60, 40, and 20 wt. % for samples prepared at 160, 180, and 200 °C, respectively, and beyond these percentages, it decreases (Figure 7e) [14,18,33]. The maximum impact strength of the MP-PP composite is 1.66 ± 0.05 kJ/m^2^ at a 200 °C molding temperature with a 20 wt. % filler concentration (Table 3).

### 3.7. Microstructure of Composites

**Figure 9:**
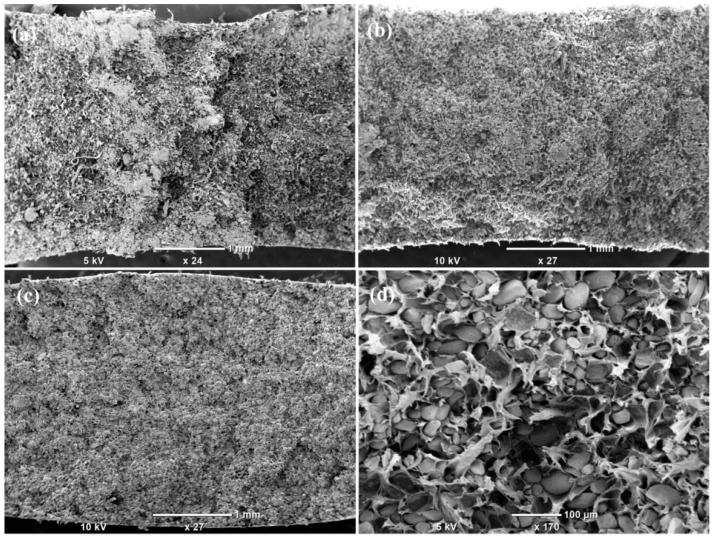
SEM micrographs of (**a**) tensile, (**b**,**d**) flexural, and (**c**) impact test-fractured MP-PP composites.

Fractured surfaces of the tensile/flexural/impact test samples are displayed in Figure 9. To study the fractured surfaces of prepared MP-PP composites, samples showing maximum tensile, flexural, and impact strength were used. Tensile and flexural strength test samples prepared at a 160 °C injection molding temperature with a 20% filler concentration were analyzed for studying the fractured surfaces. The sample prepared at a 200 °C injection molding temperature with a 20% filler concentration was analyzed for studying the microstructure of the fractured impact test sample. The SEM micrograph of the fractured tensile test specimen (Figure 9a) indicates the pullouts of polypropylene in the marble waste particulate composite and ductile failure of the sample. Regular inclusions visible in the micrographs (Figure 9a–d) correspond to marble waste. In Figure 9d, PP was perceived as a layer containing marble waste particulates, and the homogeneousness of the marble waste filler in the polymer matrix is visible in composites.

### 3.8. Thermal Conductivity of Composites

**Figure 10:**
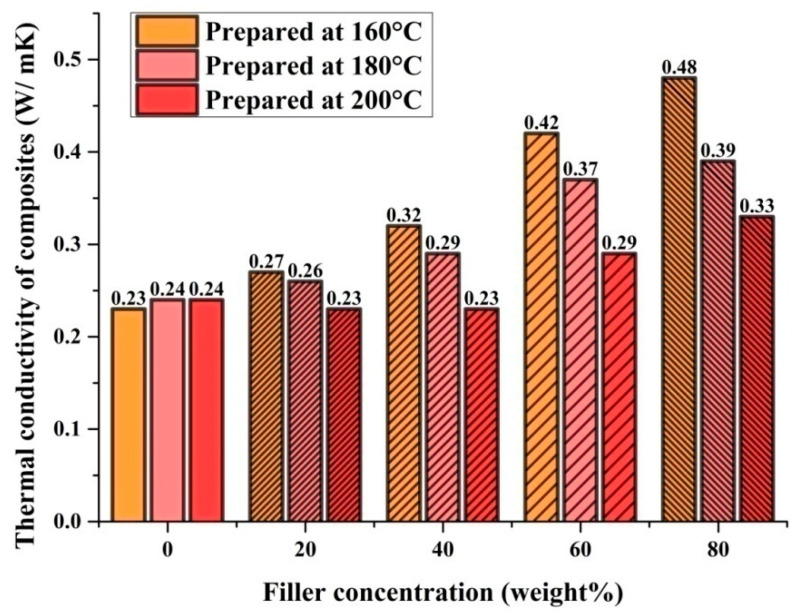
Room-temperature thermal conductivity of MP-PP composites/PP.

The thermal conductivity of pure PP and MP-PP composites has been measured as a function of filler concentration at room temperature. To determine the thermal conductivity of the fabricated composite samples as per Equation (9), three observations were taken, and their mean value is presented in Figure 10.

The thermal conductivity of pure PP was found to be 0.23 W m^−1^K^−1^ for samples fabricated at 160 °C and0.24 W m^−1^K^−1^ for samples fabricated at 180 and 200 °C. MP-PP composites prepared at 160 °C with 20, 40, 60, and 80 wt. % filler concentrations showed thermal conductivities of 0.27, 0.32, 0.42, and 0.48 W m^−1^K^−1^, respectively. MP-PP composites prepared at 180 °C with 20, 40, 60, and 80 wt. % filler concentrations showed thermal conductivities of 0.26, 0.29, 0.37, and0.39 W m^−1^K^−1^, respectively. MP-PP composites prepared at a 200 °C injection molding temperature with 20 and 40 wt. % filler concentrations showed a thermal conductivity of 0.23 W m^−1^K^−1^, and with 60 and 80 wt. % filler concentrations showed thermal conductivities of 0.29 and 0.33 W m^−1^K^−1^, respectively. The results indicated that the thermal conductivity increases with the increment in concentration of marble waste filler because the filler provides the path for heat transfer except in case of the samples prepared at an injection molding temperature of 200 °C, where the value of thermal conductivity slightly decreases for 20% and 40% filler concentrations [34,47]. It is observed that the thermal conductivity of pure PP samples slightly increases with the increase in injection molding temperature, whereas the thermal conductivity decreases in the case of the MP-PP composite samples with an increase in the injection molding temperature from160 to 200 °C. The minimum thermal conductivity value of the MP-PP composite has been observed as 0.23 W m^−1^K^−1^ at a 200 °C molding temperature with 20 and 40 wt. % filler concentrations. The maximum value of the thermal conductivity of the MP-PP composite has been observed as 0.48 W m^−1^K^−1^ at a 160 °C molding temperature with an80 wt. % filler concentration.

The results of the physicochemical analysis of marble waste particulates have indicated that this can be potential filler for the development of hybrid polymer composites for multiple applications. It is worth noting from the present study that the decrease in mechanical strength with the increase in filler content may be due to the agglomeration phenomenon of filler particles forming large clusters of filler in the polymer matrix and poor interfacial bonding between the filler and polymer. As a result, the surface contact area between the particles and the matrix decreases [48,49]. To overcome this problem, modification of the marble waste particulates may be done by suitable coupling agents [50,51,52,53,54,55]. The developed composites have shown a density varying from 0.96 to 1.27 g/cm^3^ and a very low water absorption of 0.006–0.034%. Marble waste particulates polypropylene sustainable composites have been developed with a tensile and flexural strength varying from 22.06 to 30.65 MPa and 43.27 to 58.11 MPa, respectively, for the molding temperature of 160 °C. The impact strength of the sustainable composites has been found to increase with filler concentration and the maximum impact strength was recorded as 1.66 kJ/m^2^ with a 20% filler concentration at a molding temperature of 200 °C. The thermal conductivity of particulates-reinforced sustainable composites was as low as 0.23 W m^−1^K^−1^ at a 200 °C molding temperature with 20% and 40% filler concentrations, and the maximum thermal conductivity was 0.48 W m^−1^K^−1^ at a 160 °C molding temperature with a80% filler concentration. The present study has shown that the lightweight, waterproof composite with better mechanical and thermal properties using marble waste particulates can be manufactured as a potential civil infrastructural material.

## 4. Conclusions

It is evident from this study that the moisture content, bulk density, and specific gravity of the marble waste particulates were found to be 1.80 ± 0.09%, 1.39 ± 0.01 g/cm^3^, and 2.58 ± 0.03, respectively. The mean particle size of marble waste particulates was 32.62 ± 0.61 µm and the pH was 6.58 ± 0.26. An electrical conductivity of 0.065 ± 0.02dS/m was recorded and the elemental analysis showed the presence of carbon, calcium, and magnesium as major constituents followed by a small concentration of aluminum, silicon, manganese, sodium, iron, potassium, and titanium. The XRD results confirmed the presence of quartz (SiO_2_), dolomite (CaMg(CO_3_)_2_), and calcite (CaCO_3_) mineral phases.

The MP-PP composite showed a low density composite (0.96 g/cm^3^) at 200 °C molding temperature with 20 % filler concentration, increases in density were recorded with an increase in filler concentration, and maximum density (1.27 g/cm^3^) was recorded at 160 °C molding temperature with 80% filler concentration. The water absorption of the MP-PP composite found to be very low (0.006%) at a 180 °C molding temperature with 80 %filler concentration. The tensile and flexural strength of MP-PP composite was little higher (30.65 ± 0.99 and 58.11 ± 0.68 MPa, respectively), at 160 °C molding temperature with 20% filler concentration. The impact properties of fabricated composites rose with the increment in filler concentration up to 60, 40, and 20 %for samples prepared at 160, 180, and 200 °C, respectively. The maximum impact strength of the MP-PP composite was 1.66 ± 0.05 kJ/m^2^ at 200 °C molding temperature with 20 %filler concentration. The thermal conductivity value of the MP-PP composite was observed as 0.23 W m^−1^K^−1^ at 200 °C molding temperature with 20 and 40% filler concentrations, and the maximum thermal conductivity was recorded as 0.48 W m^−1^K^−1^ at 160 °C molding temperature with 80% filler concentration.

The results reveal that marble waste particulates can be a potential filler and reinforcement material with polypropylene system for the development of lightweight, waterproof, high-strength, and low-thermally conductive hybrid polymer composites for multiple applications.

## Figures and Tables

**Table 1:** Physicochemical properties of marble waste particulates.

Parameter	Values
Moisture content (%)	1.80 ± 0.09
Mean particle size ( µm)	32.62 ± 0.61
Color	White
Texture (USDA soil textural class)	Silt
Bulk density (g/cm^3^)	1.39 ± 0.01
Specific gravity	2.58 ± 0.03
pH at 25 °C	6.58 ± 0.26
Electrical conductivity (dS/m)	0.065 ± 0.02

**Table 2:** EDS table showing the element composition of marble waste particulates.

Element	Mass %
Oxygen	39.36
Carbon	25.02
Calcium	22.04
Magnesium	7.41
Copper	2.02
Aluminum	1.33
Silicon	0.96
Zinc	0.94
Manganese	0.27
Nickel	0.27
Sodium	0.14
Iron	0.11
Potassium	0.07
Titanium	0.05
Total	100%

**Table 3:** Propertiesof MP-PP composites/PP.

Filler Concentration (wt. %)	Density (g/cm^3^)	Water Absorption (%)	TS (MPa)	TM (GPa)	FS (MPa)	FM (Gpa)	IS (kJ/m^2^)
MP-PP composites prepared at 160 °C injection molding temperature							
0	0.87 ± 0.01	0.008 ± 0.01	31.74 ± 4.25	7.47 ± 1.72	59.96 ± 0.97	1.85 ± 0.07	0.50 ± 0.16
20	0.98 ± 0.01	0.015 ± 0.01	30.65 ± 0.99	5.23 ± 0.96	58.11 ± 0.68	2.36 ± 0.12	0.56 ± 0.02
40	1.09 ± 0.01	0.019 ± 0.02	28.19 ± 1.65	5.04 ± 0.31	54.22 ± 0.63	2.81 ± 0.14	0.72 ± 0.13
60	1.18 ± 0.03	0.018 ± 0.03	26.91 ± 1.74	5.80 ± 0.24	50.53 ± 1.24	3.20 ± 0.16	0.83 ± 0.04
80	1.27 ± 0.01	0.017 ± 0.02	24.60 ± 1.16	6.20 ± 0.37	48.81 ± 0.69	3.76 ± 0.27	0.75 ± 0.04
MP-PP composites prepared at 180 °C injection molding temperature							
0	0.85 ± 0.01	0.008 ± 0.02	33.70 ± 1.02	3.43 ± 0.10	49.12 ± 0.77	1.46 ± 0.06	1.32 ± 0.08
20	0.97 ± 0.01	0.015 ± 0.01	30.06 ± 0.68	4.46 ± 0.54	53.52 ± 1.09	1.95 ± 0.14	1.40 ± 0.05
40	1.09 ± 0.01	0.026 ± 0.03	26.40 ± 0.53	4.99 ± 0.20	50.88 ± 0.47	2.52 ± 0.10	1.42 ± 0.18
60	1.18 ± 0.01	0.018 ± 0.02	22.88 ± 1.13	4.87 ± 0.79	47.64 ± 0.40	3.03 ± 0.16	0.79 ± 0.08
80	1.19 ± 0.09	0.006 ± 0.01	22.41 ± 0.21	5.99 ± 0.16	44.44 ± 0.23	3.57 ± 0.27	0.77 ± 0.01
MP-PP composites prepared at 200 °C injection molding temperature							
0	0.85 ± 0.01	0.007 ± 0.01	34.10 ± 0.89	3.26 ± 0.08	49.90 ± 0.30	1.47 ± 0.11	1.34 ± 0.10
20	0.96 ± 0.01	0.013 ± 0.01	27.62 ± 0.90	4.33 ± 0.34	49.92 ± 1.12	2.01 ± 0.09	1.66 ± 0.05
40	1.07 ± 0.01	0.034 ± 0.03	26.07 ± 1.08	5.27 ± 0.32	47.41 ± 0.42	2.45 ± 0.08	1.58 ± 0.03
60	1.18 ± 0.01	0.024 ± 0.01	23.37 ± 0.49	5.26 ± 0.09	46.38 ± 0.90	2.99 ± 0.14	0.98 ± 0.05
80	1.26 ± 0.01	0.022 ± 0.01	22.06 ± 0.70	5.67 ± 0.11	43.27 ± 0.78	3.66 ± 0.34	0.86 ± 0.03
